# Respiratory virus infection dynamics and genomic surveillance to detect seasonal influenza subtypes in wastewater: A longitudinal study in Bengaluru, India

**DOI:** 10.1371/journal.pgph.0004640

**Published:** 2025-09-12

**Authors:** Namrta Daroch, Subash K. Kannan, Vishwanath Srikantaiah, Rakesh Mishra, Farah Ishtiaq

**Affiliations:** 1 Tata Institute for Genetics and Society, Bengaluru, Karnataka, India; 2 Biome Environmental Trust, Bengaluru, Karnataka, India; Tohoku University Graduate School of Engineering School of Engineering: Tohoku Daigaku Daigakuin Kogaku Kenkyuka Kogakubu, JAPAN

## Abstract

Recent global pandemics have been caused by respiratory viruses in humans and animals with zoonotic spillover potential. Respiratory viruses, such as severe acute respiratory syndrome coronavirus 2 (SARS-CoV-2), respiratory syncytial virus (RSV), influenza A virus (IAV), and influenza B virus (IBV), share overlapping ecology and similar symptoms. However, respiratory disease surveillance is often passive, relying on clinical specimen testing. Wastewater surveillance has been used for early detection of SARS-CoV-2 variants and can differentiate between respiratory virus infections and SARS-CoV-2 peaks at the community level. In this retrospective longitudinal study covering four SARS-CoV-2 Omicron waves, we conducted monthly sampling for 28 months (812 samples) between August 2021 and December 2023 at 28 sewershed sites in Bengaluru (~11 million inhabitants), India. Using RT-qPCR kits, we quantified SARS-CoV-2 RNA concentrations, IAV, IBV, and RSV to understand community viral infections. We found 86% of samples positive for SARS-CoV-2, while positivity rates for influenza virus and RSV were lower (37% for IAV, 16% for IBV, and 15% for RSV) and this pattern was consistent across sites. We observed a seasonal increase in influenza viruses during the monsoon, peaking in October, with mean IAV viral loads of 755 copies/person/day in 2021, 2000 copies/person/day in 2022, and 1749 copies/person/day in 2023. IAV was present in January and February but absent the rest of 2022. However, IAV viral load was detected throughout 2023 (except in June). IBV showed a similar trend, peaking in October, with mean viral loads of 616.56 copies/person/day in 2021, 323.37 copies/person/day in November 2022, and 373.37 copies/person/day in September 2023. RSV displayed a shorter transmission window, peaking at around 2000 copies/person/day in October. Using genomic data, we provide evidence of changes in the relative abundance of influenza subtypes and SARS-CoV-2 variants, identifying all eight segments of influenza virus genomes and emerging SARS-CoV-2 variants in wastewater samples. Wastewater surveillance provides data on the diversity and relative abundance of respiratory viruses in urban Bengaluru that would not be reported otherwise. Under the One Health framework, wastewater surveillance can offer early warning signs and enhance traceability of infectious diseases in wildlife and humans.

## 1. Introduction

Acute respiratory illnesses (ARI) present a significant healthcare challenge in developing countries. In India, ARI is a leading cause of mortality among children under five, with considerable geographical disparities [1,2]. Improved policies and programmes are needed to reduce vaccine-preventable deaths and meet the Sustainable Development Goals for child survival targets by 2030 [2]. Furthermore, many respiratory viruses, such as SARS-CoV-2, RSV, and influenza, exhibit similar symptoms, complicating clinical diagnosis [3,4]. Given the overlapping host-virus ecology, co-infection with SARS-CoV-2 and influenza viruses in humans could alter disease transmission patterns and increase the disease burden [5].

Influenza viruses belong to the Orthomyxoviridae family and are classified into types A, B, and C. Influenza is a highly contagious viral disease caused by various strains of the influenza virus. Influenza A strains are classified into subtypes based on the combination of two proteins Haemagglutinin (HA) and Neuraminidase (NA), found on the surface of the virus. Influenza viruses undergo antigenic evolution through antigenic drift and shift in their surface glycoproteins. This requires frequent updates to influenza vaccine components and timely surveillance [6].

Birds can be infected with low pathogenic avian influenza virus (LPAI: H1-H16), and IAV isolated from or adapted to avian hosts are avian influenza viruses (AIV). Of the 16 HA subtypes identified in birds, 5 HA subtypes of IAVs (H5N1, H6N1, H7, H9N2, H11N6, H13) are known to cause human infections [7]. The H1N1 and H3N2 IAVs continue to circulate in humans as seasonal influenza. In addition to humans, IAVs have a wide range of host species, especially wild aquatic birds (e.g., Anseriformes and Charadriiformes), which are the reservoir hosts of IAVs and can cause mild disease in poultry, such as breathing problems and reduced egg production. Only some avian influenza A (H5 and H7) subtypes are classified as Highly pathogenic avian influenza virus (HPAI) which causes outbreaks and mortality in poultry and wild birds and has a nearly global distribution [8]. H5N1 subtype is a major health and economic concern due to its catastrophic impact on the poultry industry, high mortality rates, and potential as a source for a future pandemic [9].

Similarly, IBV is a seasonal virus with two main lineages, B/Yamagata and B/Victoria, which are primarily adapted to human hosts (evolved to bind specifically to receptors in the human respiratory tract) [10,11] and, to a lesser extent, to sea mammals and birds. The narrow host range limits gene assortment and subtype diversity, leading to a slower rate of antigenic shift [11]. Thus, influenza B viruses evolve more slowly than influenza A viruses. Both lineages have co-circulated worldwide in most influenza seasons since the 1980s [12]. They can coexist in different proportions within the same season and specific regions [13]. Most studies suggest that the influenza B/Victoria lineage is more prevalent than the influenza B/Yamagata lineage in younger populations [14]. However, there is limited data on the epidemiology of influenza B virus in India, except for a few clinical studies in North India exploring the co-circulation of the two lineages [e.g., 15,16]. In the post-pandemic world, the detection of influenza B/Yamagata cases has decreased globally, with a likely extinction.

RSV is divided into two major antigenic groups: Group A and Group B. Chadha *et al*. [17] reported 15.4% influenza and 5% RSV infections in a study of 16,338 suspected ARI cases.

In India, respiratory disease surveillance is inadequate due to underreporting. The Integrated Disease Surveillance Programme (IDSP) under the National Centre for Disease Control monitors the situation weekly to detect early warning signals of impending outbreaks or changes in the epidemiology of significant diseases. According to IDSP data, India experiences bimodal influenza seasons: one from January to March and another in the post-monsoon season [https://pib.gov.in/PressReleasePage.aspx?PRID=1905602]. RSV infections peak during the monsoon in September and October [18,19].

There is a need for a complementary surveillance approach independent of individual testing to identify spatial and temporal patterns in co-circulating respiratory viruses. Wastewater surveillance from selected locations to detect, quantify, and characterize infectious disease pathogens is cost-effective and useful in low-resource settings where obtaining representative data on the infectious disease burden is challenging [20].

Densely populated cities play a significant role in the spread of emerging diseases, particularly those transmitted via respiratory and faecal-oral routes. Since August 2021, our team, a collaboration between researchers from the Bangalore Life Science Cluster (BLiSc) and the city utility responsible for water supply and sewerage, has been conducting citywide wastewater surveillance of SARS-CoV-2 in Bengaluru. Through this program, wastewater samples from the influent of 28 sewage treatment plants (STPs), covering approximately 11 million inhabitants, have shown strong correlations between RNA from SARS-CoV-2 in wastewater and COVID-19 case rates in the community [21], and have characterized the spatiotemporal variation in the antibiotic resistome and corresponding bacterial community [22].

In response to the growing need to differentiate between SARS-CoV-2 outbreaks and seasonal respiratory viruses, we aimed to test wastewater surveillance to evaluate and compare RNA levels of IAV/IBV, RSV-A/RSV-B, and SARS-CoV-2 in Bengaluru. We expect these results to complement the influenza surveillance system (IDSP) and help define testing priorities and vaccination drives. This proof-of-concept study can inform guidelines for monitoring human and animal respiratory viruses. Overall, this study lays the groundwork for the systematic characterization of viral diversity in wastewater that can influence public health decision-making.

## 2. Materials and methods

### 2.1. Wastewater sample collection

**Fig 1:**
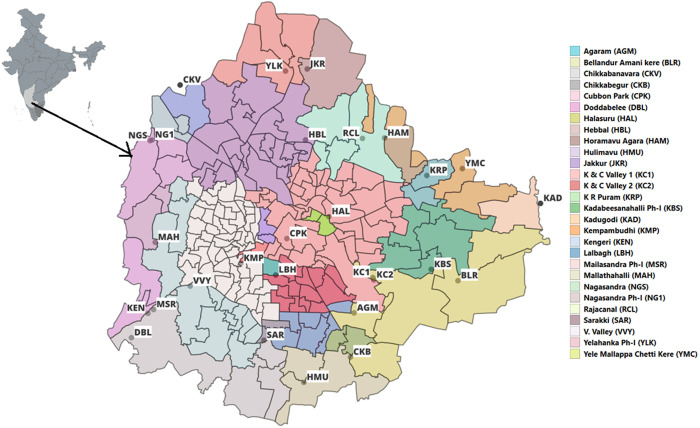
Map of Bengaluru showing the geographical locations of 28 sewershed sites screened for respiratory viruses from August 2021 to December 2023. This map was created using the *Leaflet* package (https://github.com/rstudio/leaflet) and Base map and data from the OpenStreetMap and OpenStreetMap Foundation, available under the Open Database License (https://www.openstreetmap.org/copyright).

We sampled influent wastewater from 28 centralized sewage treatment plants (STPs) in Bengaluru, India. Grab samples were collected once a month from each STP from August 2021-December 2023 between 0800–1400 h. These STPs covered different catchment area sizes: nine small (serving 10,000 − 60,000 population), nine medium (serving 100,000 – 350,000 population), and ten large (serving 400,000 − 2,480,000 population) STPs (Fig 1). Details of inflow rate, STP capacity (volume of water), and population size are provided in S1 Table.

Samples were collected in 200 mL plastic bottles, tightly sealed upon collection, and stored at 4°C in the field. All samples were processed within 24 h at a biosafety level 2 facility as described previously [21]. Samples were subjected to heat inactivation and incubated in a 200 mL bottle at 60°C for 90 min, then divided into three replicates. Subsequently, 40 mL of the master sample (a total of 120 mL from the 200 mL bottle) was transferred to three 50 mL centrifuge tubes containing 0.9 g NaCl and 4 g polyethylene glycol (PEG; 8000 MW). The PEG and NaCl mixture were vortexed until dissolved and centrifuged at 11,000 rpm for 30 min at 4°C. After discarding the supernatant, 600 µL of Qiagen buffer AVL viral lysis buffer was added to resuspend the pellets. The solution was transferred to a centrifuge tube and briefly vortexed to dissolve the pellet. Viral RNA was extracted using the Qiagen Viral RNA Mini Kit protocol, following the manufacturer’s instructions, and 50 µL of the elution was stored at -80°C until subsequent analysis.

### 2.2. Viral Reverse Transcriptase-quantitative PCR (RT-qPCR)

We used two RT-qPCR kits to screen for SARS-CoV-2, RSV, and Influenza viruses in wastewater. For SARS-COV-2, the *Gene*Path Dx CoViDx One v2.1.1TK quantitative multiplex RT-qPCR kit was used. The kit targets three viral genes: N-gene, RdRp-gene, and E-gene, along with a human control gene (RNAase P gene) [21]. The COVID-19 Viral Load Calculation Tool (RUO; https://coviquant.genepathdx.com/) was used to quantify viral load in the samples. The tool uses Ct value cut-offs <35 with three standards of high (5000 copies/μL), medium (500/μL), and low (50 copies/μL) to calculate viral copies/μl of each sample.

For the detection of RSV-A/B (M gene), Influenza A (M gene), and Influenza B (non-structural gene), the *GenePath* Dx Multiplex kit with a human control gene (RNase P gene) was used. The test can detect H1N1 and H3N2 of Influenza A and Victoria and Yamagata strains of Influenza B. However, the kit does not distinguish between IAV and IBV or their subtypes. To quantify IAV and IBV in wastewater, RT-qPCR was carried out using the CDC FluSC2 assay (https://archive.cdc.gov/www_cdc_gov/coronavirus/2019-ncov/lab/multiplex.html). Final concentrations of 0.2 μM were used for the forward and reverse primers and 0.2 μM for the probe. A total volume of 15 μl containing 5 μl of eluted RNA and 3.75 μl of Luna Probe One-Step RT-qPCR 4X Mix with UDG (NEB, Catalog no: M3019L) was prepared under the following cycling conditions: 25 °C for 30 s, 55 °C for 10 min, 95 °C for 1 min, and 45 cycles of 95 °C for 10 s, then 56 °C for 1 min on a QuantStudio 5 Real-Time PCR System. The fluorescent signal was measured during the annealing step. Primer sequences and concentrations of primers and probes used are available in S1 File. Samples were run in duplicate wells and included negative extraction control and no template control. Samples were considered positive if Ct values were ≤35.

To determine the copy numbers of IAV (M-gene) and IBV (NS-gene), a standard curve of the linearized pUC57 plasmid was used (see S1 File for details). Only samples with negative and invalid results were tested using Pepper mild mottle virus (PMMoV) as an indicator of human fecal contamination.

### 2.3. Viral load modelling

We used consolidated data for each month to estimate changes in viral concentrations. We calculated the daily viral load, *VL* (copies/person/day), for SARS-CoV-2, influenza A, influenza B, and RSV by normalizing raw viral load, *C* (copies/mL), to the average daily STP flow, *Q(L/d),* using equation 1 [23], and *P* is the population of inhabitants in the catchment of the respective sewershed site.


$$VL=\frac{C\times Q\times 10^{3}}{P}$$ (1)

To estimate the association between viral concentrations of influenza A, influenza B, and RSV, we used Kendall’s tau (*τ*) statistic [24], as the Shapiro-Wilk normality test showed the data was not normally distributed (W = 0.54, *p* < 2.2e-16). If the coefficient is between -1 and 0, the correlation is negative; if it is between 0 and 1, it is positive. If the result is exactly 0, there is no correlation. We performed six pairwise tests, and tau values were considered significantly different from zero when *p* < 0.00009.

### 2.4. Genomic surveillance

#### (i) Metagenomic shotgun sequencing for influenza.

An unbiased metagenomic approach was used to genetically characterise pan-viral diversity [25]. Here, we analysed data for influenza viruses, and results for other viruses will be reported elsewhere. A subset of 242 grab samples, which included 10 samples for each month, both positive and negative for influenza (S2 and S3 Tables), was selected for sequencing at the Next Generation Genomics Facility in BLiSc. RNA extraction was quantified using a Qubit Fluorometer (Invitrogen) with a Qubit HS RNA assay kit. RNA integrity was analyzed using a 4200 Tape Station (Agilent) and an RNA Screen Tape assay kit. To enrich the viral RNA content before library preparation, ribosomal RNA (rRNA) was depleted using a combination of two kits: NEBNext rRNA Depletion Kit v2 (Human/Mouse/Rat) (Cat No. NEB E7405) and NEBNext rRNA Depletion Kit (Bacteria) (Cat No. NEB E7860), along with RNA Sample Purification Beads (Cat No. NEB #E7405) as per the manufacturer’s protocols, starting with a total RNA concentration of 100 ng - 1μg. rRNA-depleted RNA libraries were prepared using NEBNext Ultra II Directional RNA Library Prep (Cat. No. E7765L) with Sample Purification Beads and sequenced on the NovaSeq 6000 platform using an S4 flow cell and a 2x150 bp read length kit (Illumina).

#### (ii) Targeted sequencing of SARS-CoV-2.

Libraries were prepared using the Illumina COVIDSeq Test kit (Cat No: 20043675, Illumina Inc, USA). RNA samples were primed with random hexamers for reverse transcription. cDNA) products were amplified using the ARTIC V3 and ARTIC V5.3.2 primer set targeting the entire SARS-CoV-2 genome and human cDNA targets in two multiplex PCR reactions. The amplified product was processed for tagmentation and adapter ligation using IDT for Illumina Nextera DNA Unique Dual Indexes Sets A–D and IDT for Illumina-PCR Indexes Sets 1–4 (384 Indexes, Cat no: 20043137, Illumina Inc., USA). Further enrichment and clean-up were performed according to the manufacturer’s instructions.

Pooled libraries were quantified using a Qubit 4.0 fluorometer (Invitrogen, USA), and library sizes were analysed using a TapeStation 4200 (Agilent, USA). The libraries were normalised to 2 nM and denatured with 0.1 N NaOH.

The denatured libraries were sequenced at appropriate concentrations depending on the sequencing system, including the HiSeq 2500 with a Rapid SR flow cell (v2: 1 × 50 bp), the MiSeq with a flow cell (2 × 75 bp), and the NovaSeq 6000 with an SP flow cell (2 × 100 bp), following the manufacturer’s instructions (Illumina Inc.).

### 2.5. Bioinformatics

#### (i) Influenza read detection and subtype assignment.

We used two bioinformatics pipelines to detect influenza reads and assign subtypes. The first pipeline performed reference-based assembly using RefSeq genomes of influenza viruses (A, B, C). Demultiplexed raw FASTQ files were processed using Trimmomatic (v.0.39) [26] (LEADING:3 TRAILING:3 SLIDINGWINDOW:4:30 MINLEN:50) to remove Illumina adapters. Potential human reads were removed using the Kraken Human database (Kraken v.2.1.3) [27]. Paired-end trimmed reads were re-paired with the repair.sh utility from bbmap (sourceforge.net/projects/bbmap/). Trimmed FASTQs were mapped to all influenza whole genome sequences available in the Influenza Resource Database (IRD) of the National Centre for Biotechnology Information (NCBI). Bowtie2 (v.2.2.5) [28] was used to map quality-controlled reads to the influenza database using the influenza.fasta file https://ftp.ncbi.nih.gov/genomes/INFLUENZA/influenza.fna (downloaded on 08.04.2023). SAMtools coverage/bedcov [29] was used to calculate basic stats of the mapped file, resulting in read count calculations for each subtype and read length. Bam ‘*validate*’ was used to calculate the mapping rate of each sample. Sample metadata from NCBI was used to annotate likely subtypes and hosts of influenza virus sequences detected in each collection location, based on the closest match in the NCBI. To avoid false positives resulting from index swapping during capture [30,31], we established a threshold of 0.1% of the total reads for each virus in the appropriate pool. This was based on published studies and is consistent with our experiences with dual-indexed sequencing libraries and the HiSeq 2500 platform. Samples were required to have a greater percentage of reads assigned to a particular virus than the percentage of reads assigned to that virus across all batch-specific controls. Read counts were further normalized as reads per million (RPM) of total (trimmed) reads. To reduce false-positive virus discovery, an arbitrary acceptance criterion of viruses with threshold of RPM > 1 for a significant metagenomic NGS findings was considered [32].

The second pipeline used the publicly available 981,537 iav_serotype (v0.1.4) software package (https://github.com/mtisza1/influenza_a_serotype). Paired-end reads were quality filtered using fastp (v0.23.4) with default settings. Reads were aligned to the Influenza_A_segment_sequences database v1.1 using minimap23 (v2.28-r1209) with flags “-cx sr --secondary=yes -f 100000” to allow secondary alignments. Average nucleotide identity (ANI) and alignment fraction (AF) were assigned to a serotype if the best alignment was exclusive to one serotype and (ANI*AF >= 0.9); otherwise, reads were assigned as “ambiguous”.

Data processing and visualization were implemented in RStudio (v.4.2.1) [33], using multiple packages (*ggplot2*, *tidyverse*, *corrplot*, *Hmisc*).

#### (ii) SARS-CoV-2 variant detection.

Wastewater samples contain a mixture of variants, unlike clinical samples that may have a single variant. Raw reads were aligned with the SARS-CoV-2 reference genome (MN908947) using BWA Mem [34]. Coverage statistics were obtained with SAMtools coverage/bedcov [29]. The alignment facilitated single nucleotide variant (SNV) calling via iVar [35], which trimmed primers and produced mutation frequency data and adjusted p-values (Fisher’s test) for altered positions from the BAM files. iVar was run with a minimum base quality filter of 20 using the SARS-CoV-2 reference genome and the feature file Sars_cov_2.ASM985889v3.101.gff3 from NCBI. To predict lineage abundances, a deconvolution matrix was generated using *Freyja* (https://github.com/andersen-lab/Freyja) [36], a bioinformatic pipeline for wastewater analysis. Based on SNV frequency and sequencing depth measurements, *Freyja* estimates the true lineage abundances in the sample, using the UShER tree with WHO designation and outbreak.info metadata [37].

## 3. Results

RNA from all target viruses was detected in wastewater. SARS-CoV-2 was found in 86% (699/812) of samples, significantly more than the other viruses. IAV and IBV were detected in 37% (305/812) and 16% (131/812) of samples, respectively, while RSV was found in 15% (123/812). There were 48 samples positive for all three viral targets, and 127 samples were positive for both IAV and IBV.

**Fig 2:**
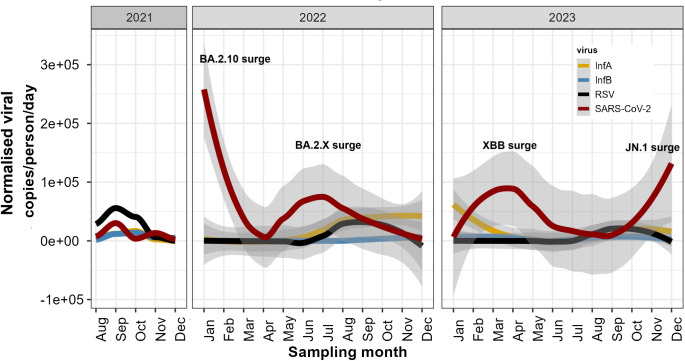
Temporal trends of respiratory viruses in untreated wastewater. Raw viral load is normalised by flow rate and population of each sewershed site. The grey band around the coloured lines shows confidence intervals for each viral target.

The analysis of the influenza virus showed that IAV predominated over IBV and RSV. There was a seasonal increase in influenza viruses during the monsoon, peaking in October with mean viral loads of 755 copies/person/day in 2021, 2000 copies/person/day in 2022, and 1749 copies/person/day. IAV was present in January and February but was nearly absent for the rest of 2022. However, IAV was detected throughout 2023 (except in June). IBV followed a similar trend, peaking in October with mean viral loads of 616.56 copies/person/day in 2021, 323.37 copies/person/day in November 2022, and 373.37 copies/person/day in September 2023. RSV peaked at around 2000 copies/person/day in October (Fig 2; S4 Table).

**Fig 3:**
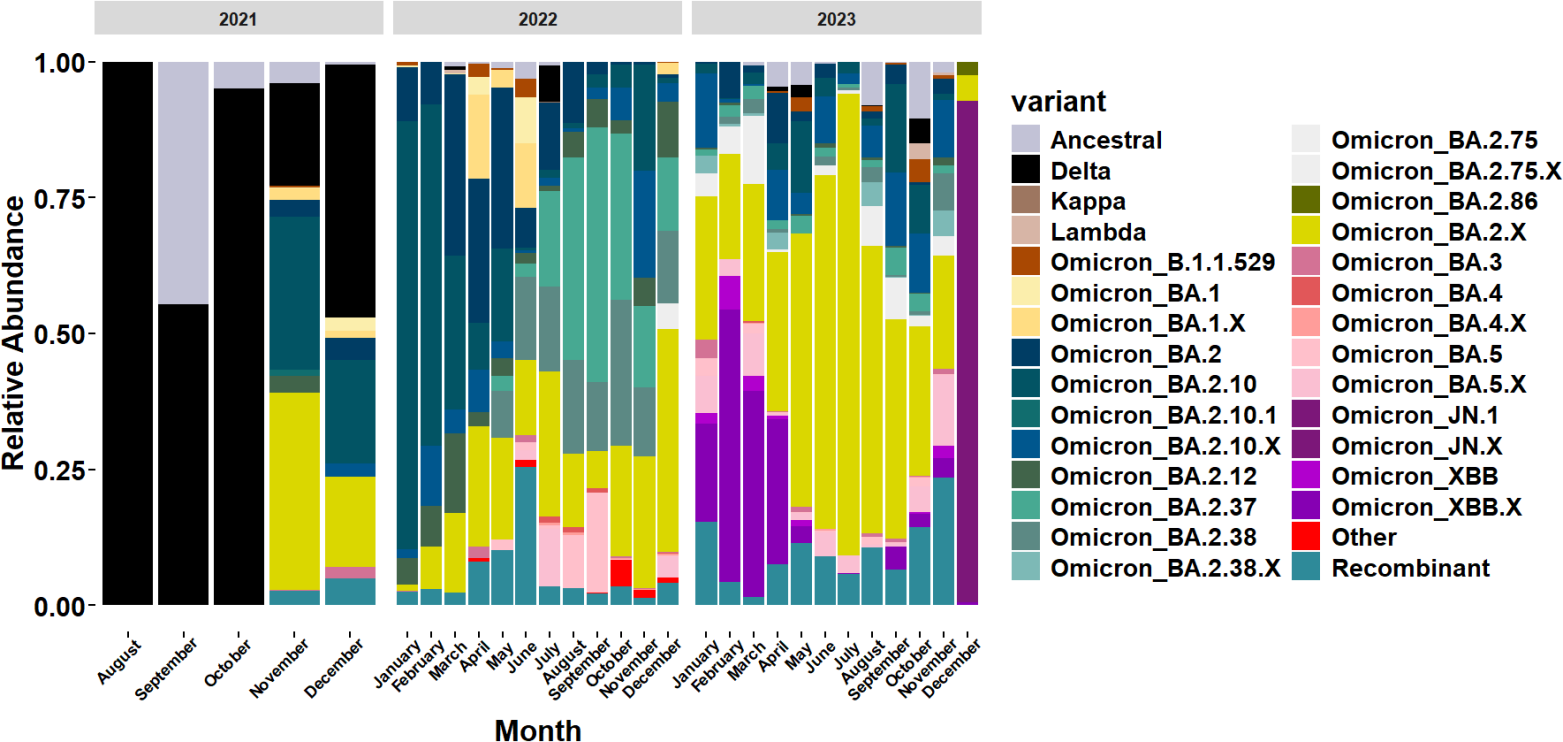
Temporal dynamics of SARS-CoV-2 variant diversity in wastewater in Bengaluru.

SARS-CoV-2 was prevalent throughout the study period, with four main surges identified in wastewater genomic data: BA.2.10, BA.2.X, XBB, and JN.1. While IAV and RSV followed a seasonal pattern, SARS-CoV-2 was driven by the emergence of new variants (Fig 3). From August 2021 to December 2021, Delta and ancestral SARS-CoV-2 lineages dominated. Starting in November 2021, there was an exponential increase in Omicron lineages—BA.1, BA.2.10, BA.2.X, and BA.3. In January 2022, BA.2.10 was the dominant lineage, which later transitioned into BA.2.X lineages driving viral loads in June 2022. In January 2023, the XBB variant was detected in wastewater, peaking in the first week of April 2023. Starting of JN.1 surge was observed in the last week of December 2023. There was no overlap in SARS-CoV-2 surges and IAV, IBV, and RSV peaks, except for the XBB surge, which overlapped with IAV and IBV in 2023. This was further supported by Kendall tau statistics. SARS-CoV-2 RNA showed no significant correlation with any viral target. However, IAV, IBV, and RSV exhibited significant cross-correlation, with tau values ranging from 0.46 (IAV and IBV) to 0.23 (IAV and RSV) and 0.21 (IBV and RSV) (S5 Table).

**Fig 4:**
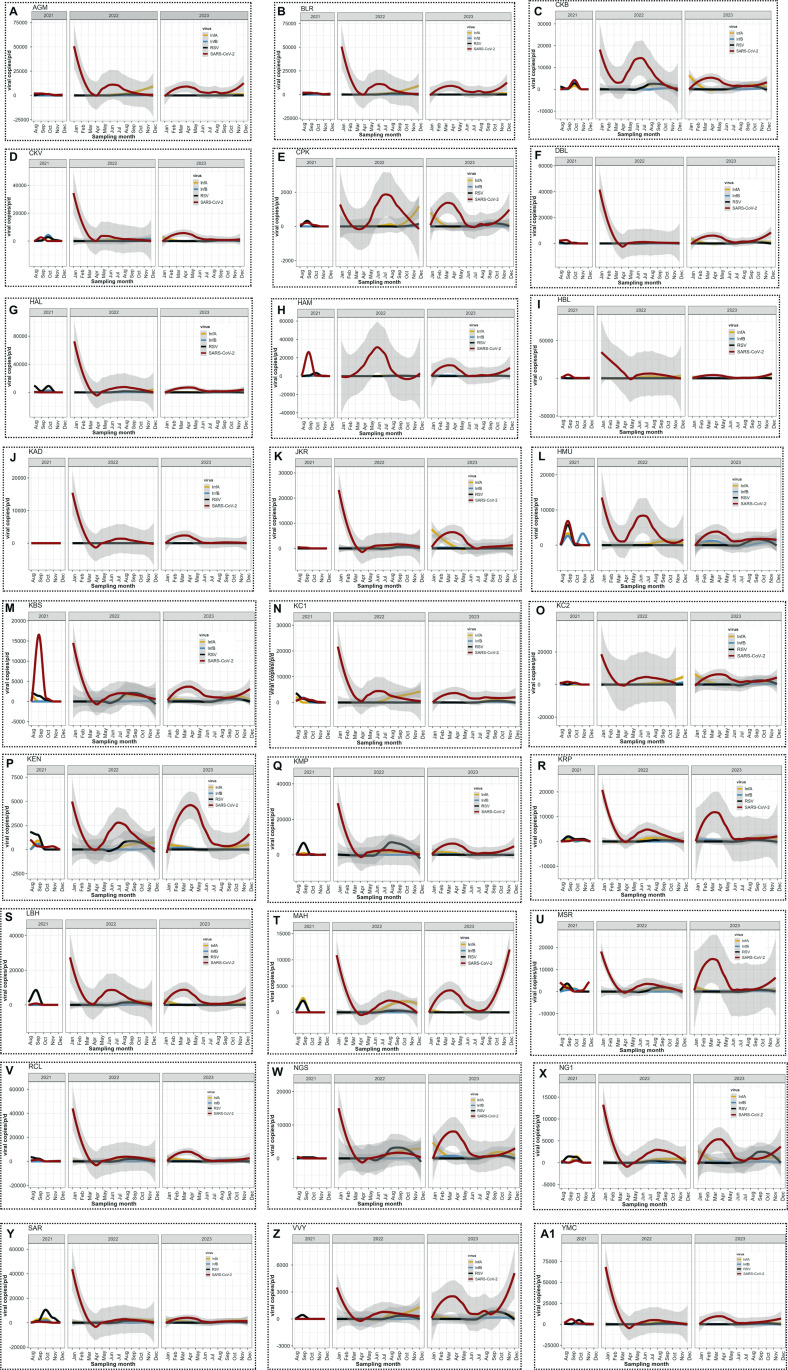
Temporal dynamics of normalized viral load (gene copies/person/day) of SARS-CoV-2, IAV, IBV, and RSV across sewershed sites. The grey band around the coloured lines shows confidence intervals for each viral target.

SARS-CoV-2 viral trend lines showed varying trajectories across sewershed sites (Fig 4). The viral load remained low in 2021 (Fig 4). In the last week of December 2021, the BA.2.10 surge began, recorded in 28 sites, while the BA.2.X surge was not uniformly detected. The XBB surge occurred at all sites with a higher magnitude (~7x10^6^ copies/person/day) compared to the BA.2.10 surge (~5.6x10^5^ copies/person/day).

Similarly, IAV, IBV, and RSV viral trend lines were recorded in AGM, BLR, CKB, CPK, JKR, KC1, KC2, MAH, NG1, NGS, and VVY in 2022. AGM had the highest IAV viral load in 2022 (2267 copies/person/day), followed by HBL (1064.33 copies/person/day) and NGS (1002.91 copies/person/day). In 2023, viral loads included BLR (1600.25 copies/person/day), CKB (1391. 40 copies/person/day), JKR (1438.55 copies/person/day), KC1 (1359.79 copies/person/day), NGS (1232. 24 copies/person/day), and SAR (1186 copies/person/day). RSV was dominant in 2021 in CKB, CKV, HMU, HAL, HAM, KEN, KMP, KRP, LBH, MAH, MSR, NG1, and SAR, and was recorded in 2022 in CKB, KBS, KEN, KMP, MSR, and NGS (S6 Table).

### 3.1. Influenza virus read abundance and alignment to genome segments

We analysed a subset of 242 wastewater samples from August 2021 to December 2023; details on total reads and sequencing depth are in S4 and S5 Tables. Genome coverage for all influenza virus analyses, ranged from 1.3% to 33%. The IAV reads in wastewater samples ranged from 1 to 86, with 2405 sequences detected and no single sample producing reads for every segment (S1 Fig). Reads aligned to all eight genome segments and were generally biased towards segment 1 [(PB2 = 369 (IAV); 6 (IBV); 10 (Inf C)], segment 3 [PA = 1702 (IAV); 12 (IBV)], and segment 7 [(M = 137 (IAV); 6 (IBV); 6 (Inf C)] (Fig 3). The segment 2 [PB1 = 19 (IAV); 2 (IBV);16 (Inf C)], segment 4 [HA = 14 (IAV); 11 (IBV); 10 (Inf C)], segment 5 [NP = 15 (IAV); 4 (IBC);10 (Inf C)], segment 6 [NA = 2 (IAV); 2 (Inf C)], segment 8 [NS = 4 (IAV); 4 (IBV); 30 (Inf C)] had > 20 matches in the database. Additionally, influenza C had hemagglutinin-esterase (HE) gene = 4 and P3 gene = 2. The mean read number and coverage were 7 ⋅ 6% for the HA segment and 10.9% for the NA segment.

**Fig 5:**
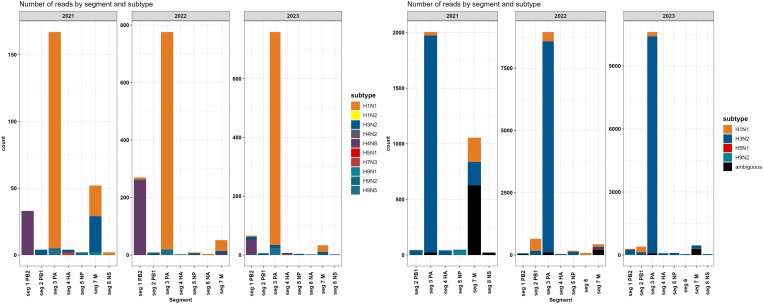
Number of reads amplified and sequenced from wastewater that matched the influenza virus whole genome database using two bioinformatic pipelines: left: NCBI RefSeq Influenza genomes; right: iav_serotype.

Similarly, the iav_serotype pipeline assigned reads to all eight segments of the influenza A genome, with a bias towards segments 3 and 7 (Fig 5). However, the iav_serotype software tends to take a conservative approach that can obscure minority strains; read alignments were assigned to a particular subtype if the best alignment was exclusive to one subtype and ANI*AF >=0.9. Using iav_serotype, the reads were assigned to the seasonal influenza subtypes H3N2 and H1N1.

### 3.2. Influenza diversity in wastewater

**Fig 6:**
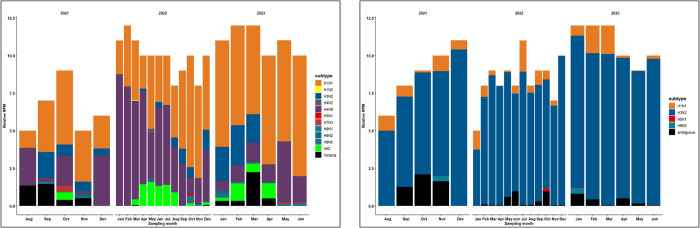
Temporal variation in the relative abundance of influenza subtypes in Bengaluru sewage using two bioinformatic pipelines: left: NCBI RefSeq Influenza genomes; right: iav_serotype.

Using RefSeq alignments, wastewater sequences identified ten influenza A subtypes (H1N1, H1N2, H3N2, H4N8, H5N1, H7N3, H9N1, H9N2, and H9N5), one influenza B subtype (Victoria), and influenza C (Fig 6). H1N1 and H4N8 were the dominant subtypes, followed by H3N2.

Using the iav_serotype pipeline, the reads were aligned to all eight segments representing the metagenomes of influenza subtypes in a single sample, reflecting the true diversity of influenza viruses in wastewater, that would otherwise remain undetected by targeted sequencing or RT-PCR. Nonetheless, the patterns in influenza A subtypes H1N1 and H3N2 correspond with the RT-PCR viral load.

**Fig 7:**
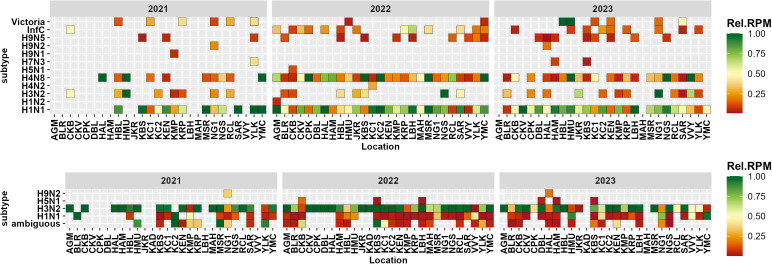
Top: Influenza detection and subtype assignment using RefSeq genomes of influenza viruses (A, B, C) revealed diversity in influenza A (InfA: H1N1, H1N2, H3N2, H4N2, H5N1, H7N3, H9N1, H9N2, H9N5), influenza B (InfB: Victoria), and influenza C (InfC) subtypes across sewershed sites. KAD had no assigned reads. Bottom: Influenza detection using the iav_serotype pipeline identified H1N1, H3N2, H5N1, and H9N2 subtypes.

**Fig 8:**
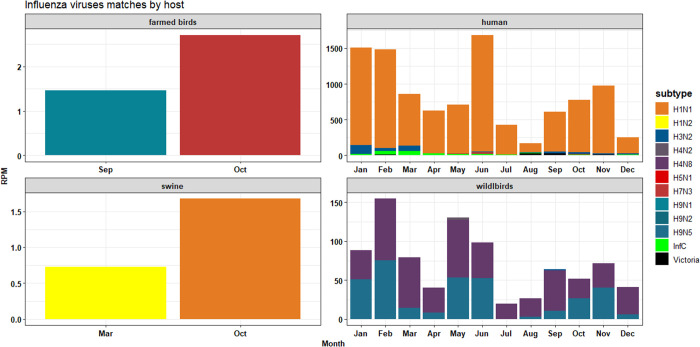
Proportion of unique reads from samples assigned similarity to human-like and avian-like influenza subtypes based on the NCBI database.

The relative abundance of these subtypes varied with space and time (Fig 7). Using RefSeq genomes, H1N1 was the dominant subtype at all sites, followed by H4N8, except at KAD. The second pipeline using iav_serotype software revealed H1N1 and H3N2 as the main subtypes, with other subtypes assigned as ambiguous. H5N1 reads were found using both pipelines in the same sample in October 2022. Furthermore, the number of reads for the human-like influenza subtype (H1N1) was larger than for the avian-like subtypes (H4N8 and H9N5) (Fig 8).

## 4. Discussion

Wastewater surveillance, or environmental surveillance, has proven to be a valuable epidemiological tool for monitoring respiratory viruses such as SARS-CoV-2 in both sewered and non-sewered communities [38–40]. In India, environmental surveillance of poliovirus (Global Polio Eradication Initiative, e.g., [41]) played a crucial role in poliovirus eradication in 2012. In a multi-city partnership (Bengaluru, Pune, Hyderabad), wastewater surveillance was used to track the evolution of the COVID-19 pandemic [21,42,43]. The success of wastewater surveillance extends beyond merely detecting the pathogen; it also includes the potential usefulness of wastewater data in informing public health actions [44]. The effectiveness of this community-level surveillance method often depends on having a well-mapped sewage network and the ability to identify hotspots for interventions (e.g., vaccination, public awareness programs).

As the COVID-19 public health emergency recedes, existing wastewater surveillance systems can be expanded to identify endemic and re-emerging infections (e.g., cholera, hepatitis, non-polio viruses, influenza) [45–47]. A short-term study in community and hospital wastewater treatment plants by Panneerselvam *et al*. [48] correlated influenza virus positivity in wastewater with clinical cases. However, there is a need to combine RT-qPCR with wastewater genomic epidemiology to fully understand the diversity of influenza subtypes at the community level, how wastewater data can inform outbreaks in the absence of clinical testing, and how the emergence of new variants drives surges in viral load across sewershed sites.

In this longitudinal study, conducted from August 2021 to June 2023 at 28 centralized sewershed sites in Bengaluru (~11 million inhabitants), demonstrates that wastewater surveillance effectively monitors infectious diseases and transmission dynamics of respiratory viruses beyond SARS-CoV-2. Wastewater data on respiratory virus abundance in Bengaluru provided new insights not available through clinical surveillance and can identify circulating strains associated with poultry and other farmed birds.

Using an untargeted metagenomic approach on wastewater samples, we determined various subtypes of influenza viruses, showing concordance between RT-qPCR and read counts. However, RT-qPCR is optimized to assess the presence and absence of target genes based on infection severity and does not capture signals below a certain threshold, i.e., low viral load. In contrast, Illumina sequencing can confirm that presence of a pathogen with accuracies and sensitivities as low as one read to confirm the presence of a pathogen and has been shown to be highly comparable to commercial viral panels (e.g., Graf *et al*. [49]). The unbiased nature of sequencing allows us to query a theoretically unlimited number of pathogens in parallel, increasing the chance of detecting all pathogens, including human viruses, and resulting in a higher positivity rate. We used sequencing reads from samples with low read counts (producing <3 reads per subtype, below our arbitrary significance level of RPM > 1). Our results demonstrate that untargeted metagenomics can supplement PCR-based tests and that wastewater metagenomic data can investigate viral etiologies of respiratory infections in low-resource settings in two contexts: (i) to understand the pathogen driving seasonal illness patterns at a community level, revealing significant diversity in IAVs dominated by H1N1, H4N8, and H3N2, while low RSV and IBV viral loads resulted in limited reads and the absence of IBV/Yamagata; (ii) to help define testing priorities and vaccination drives at a city level. The seasonality of influenza varies within India and often complicates appropriate vaccination recommendations, particularly the timing of vaccination campaigns in tropical regions [50].

We used two analytical pipelines with limitations: the reference-based assembly using RefSeq genomes of influenza viruses (A, B, C) had a poor mapping rate (S5 Table), while the iav_serotype pipeline applied a stringent criterion for assigning serotypes, requiring the best alignment to be exclusive to one serotype and have an ANI*AF of at least 0.9. This second approach limited the expected diversity in wastewater data. This underscores the need for an amplicon-based targeted whole-genome sequencing approach (e.g., ARTIC methodologies for SARS-CoV-2 sequencing) to improve genomic coverage and subtype detection across the segmented influenza genome.

We detected dynamic signals of IAV and IBV in wastewater across sewershed sites. The SARS-CoV-2 viral trend varied among sewershed sites in Bengaluru. Daniel *et al*. [51] found that large and medium-sized sewershed sites offered city-level trend insights, while small sites were more effective during low-prevalence periods. Viral shedding levels, site-specific differences in physical characteristics (catchment size), in-sewer travel time, and runoff from heavy rain could significantly influence virus detectability and contribute to variability in lead time at STP [52]. Additionally, sampling frequency (daily versus weekly) and sample type (grab versus composite) impact the resolution and quality of information from the wastewater system [53]. For instance, weekly sampling of geographically dispersed wastewater treatment plants is adequate to monitor broader community trends, while monthly sampling indicates the presence of the virus in the sewershed. Our proof-of-concept study using monthly sampling and normalised data by population size and flow rate effectively captures seasonal trends in viral load. However, for studies comparing positivity with clinical data [e.g., 21,48,51], weekly sampling will provide robust actionable data.

While there is no clinical data from Bengaluru to compare these patterns, wastewater data aligns with regional trends in influenza positivity in Karnataka (Bengaluru Medical College and Research Institute) from October to November 2021 and July to September 2022 [54].

Wastewater data facilitates epidemiologic investigations and vaccine effectiveness studies. Real-time sequencing of wastewater for influenza will improve surveillance, enable early detection of antiviral drug resistance (reducing indiscriminate antibiotic use), and guide vaccination strategies. Integrating wastewater data with clinical surveillance will provide a comprehensive view of disease prevalence in the community.

Changes in human behaviour driven by non-pharmaceutical interventions (e.g., masking, handwashing, reduced movement) during COVID-19 reshaped the evolution and distribution of seasonal influenza viruses [55,56]. Eden *et al*. [57] recorded reduced genetic diversity in RSV during the acute phase of the COVID-19 pandemic and extensive outbreaks in early 2021 following the relaxation of COVID-19 control measures. However, the evidence from India is limited [58] where the decline in influenza incidence is associated with COVID-19-related disruptions, poor health-seeking behavior, and reduced testing due to overburdened health systems, resulting in fewer influenza cases rather than a true reduction in disease transmission [59]. The absence of influenza B/Yamagata further supports the trend observed elsewhere as a possible extinction during the pandemic. This emphasises the importance of robust and timely surveillance data to inform vaccine component selection, and continued surveillance is needed to assess whether these changes will lead to sustained seasonal shifts in the post-pandemic years.

Cities offer a heterogeneous, dynamic environment, interspersed with pet markets, poultry, and livestock. The blurred boundaries between rural and urban areas, along with dense populations, create potential hotspots for emerging infectious diseases (e.g., severe acute respiratory syndrome (SARS) and avian flu). The presence of avian-like H4N8 and H9N2 underscores the need for a One Health approach to influenza surveillance. Wastewater data can help identify potential links and transmission drivers (e.g., increasing human and poultry populations, movements, and inadequate biosafety and biosecurity practices in backyard poultry farms and markets) to guide disease control strategies. Addressing this issue requires multisectoral collaboration between human and veterinary health sectors, along with the exchange of surveillance data to inform policy.

## 5. Conclusion

We screened 812 wastewater samples from 28 sewershed sites in Bengaluru between August 1, 2021, and December 31, 2023. Our findings show that wastewater surveillance can effectively detect and track SARS-CoV-2 infections, variant diversity, and shifts in influenza virus transmission using a single community-level sampling strategy. This approach is more economical than clinical testing and does not require clinician-led sampling. We observed a seasonal increase in influenza viruses during the monsoon, peaking in October, while RSV had a shorter infection window, also peaking in October, consistent with published clinical data. Viral trends varied across sewershed sites. Near real-time genomic surveillance is essential for understanding emerging patterns in viral load and informing testing priorities and vaccination strategies to assess the efficacy of health policies and non-pharmaceutical interventions. This proof-of-concept study can help develop guidelines for monitoring human and animal respiratory viruses.

## Supporting information

S1 File Preparation of Influenza A virus (IAV) and Influenza B virus (IBV) standards and quantification. (DOCX)

S1 Table Details of sewage treatment plants in Bengaluru. (XLSX)

S2 Table Influenza read detection and subtype assignment using RefSeq Genomes. (XLSX)

S3 Table Influenza read detection and subtype assignment using iav_serotype pipeline. (XLSX)

S4 Table Temporal dynamics in viral load. (XLSX)

S5 Table Kendall’s test of association between wastewater concentrations of viral RNA. (XLSX)

S6 Table Mean viral load (copies/person/day) for each sewershed site. (XLSX)

S1 Fig Influenza A subtypes detected in wastewater using virome sequencing. Panels show dereplicated reads from virome sequencing aligned to influenza A genomes in the NCBI database and the distribution of influenza subtype reads across samples.
(PNG)
