# Supplementary material for: Respiratory virus infection dynamics and genomic surveillance to detect seasonal influenza subtypes in wastewater: A longitudinal study in Bengaluru, India
Source: PLOS Glob Public Health. 2025 Sep 12;5(9):e0004640. doi: 10.1371/journal.pgph.0004640 (PMC12431432; doi:10.1371/journal.pgph.0004640)
Supplement: S1 File — (DOCX) [file pgph.0004640.s001.docx]

**Preparation of Influenza A virus (IAV) and Influenza B virus (IBV) standards and quantification**

**Preparation of quantification standards**

The synthetization of plasmid cassette was generated commercially by Genscript on the IAV and IBV sequences. The 300 bp long DNA string of IAV included the priming sites for the primers and probes of the IAV (M-gene; InfA) target sequences (WHO 2021; CDC 2009) and the 228 bp long DNA string of IBV (NS-gene; InfB). The DNA string was cloned into a pUC57 plasmid vector (Table A).

**Estimate of IAV and IBV Plasmid DNA copy number**

First, the plasmid DNA was quantified using the Qubit DNA High Sensitivity assay. To calculate the plasmid DNA copy number, 0.5μl of plasmid of IAV and IBV was dissolved in 50 μl of nuclease free water (Table A).

**Table A: Plasmid concentration details**

| Target | Qubit High Sensitivity DNA concentration (ng/µL) | Average Qubit High Sensitivity DNA concentration (ng/µL) |
| --- | --- | --- |
| IAV plasmid DNA | 0.628 | 0.622 |
|  | 0.617 |  |
| IBV plasmid DNA | 0.703 | 0.694 |
|  | 0.686 |  |

For an accurate estimation of the plasmid copy numbers, we used QIAcuity Digital PCR System (hereafter dPCR). First, we pooled both IAV and IBV in equal concentration as determined by Qubit assay (Table A). To quantify the plasmid DNA copy number, seven consecutive 10-fold serial dilutions (dilution 1- dilution 7) were prepared using nuclease free water and among this dilution 4 (D4) dilution was selected for dPCR (Table B). Dilutions with DNA quantities exceeding 10,000 copies/µl were not included to avoid saturation and maintain linearity, with the highest concentration was selected as <10^4^ copies/ul. Therefore, D4 was selected for dPCR quantification to ensure precise and cost-effective measurement with the detection range of the dPCR.

Subsequently, the copy numbers for the remaining 10- fold serial dilutions were back calculated based on the D4 dilution and IAV and IBV plasmid DNA copy numbers were determined (Table B).

**Table B: IAV and IBV plasmid DNA copies count for seven consecutive 10-fold serial fold dilutions based on D4-dPCR**

| Dilution | Volume of Plasmid DNA  (µL) | Volume of Nuclease-free water (µL) | Total volume (µL) | Fold dilution | *Back-calculated copies number using D4 dPCR data |
| --- | --- | --- | --- | --- | --- |
| D1 | **4.24 IAV + 3.8 IBV** | **392** | **400** |  | **IAV=5732000 copies/ 5** µL |
|  |  |  |  |  | **IBV=5804000 copies/5** µL |
| D2 | **50** µL of D1 | **450** | **500** | **10** | **IAV=573200 copies/ 5** µL |
|  |  |  |  |  | **IBV=580400 copies/5** µL |
| D3 | **50** µL of D2 | **450** | **500** | **10** | **IAV=57320 copies/ 5** µL |
|  |  |  |  |  | **IBV=58040 copies/5** µL |
| D4* | **50** µL of D3 | **450** | **500** | **10** | **IAV=5732 copies/ 5** µL |
|  |  |  |  |  | **IBV=5804 copies/5** µL |
| D5 | **50** µL of D4 | **450** | **500** | **10** | **IAV=573.2 copies/ 5** µL |
|  |  |  |  |  | **IBV=580.4 copies/5** µL |
| D6 | **50** µL of D5 | **450** | **500** | **10** | **IAV=57.32 copies/ 5** µL |
|  |  |  |  |  | **IBV=58.04 copies/5** µL |
| D7 | **50** µL of D6 | **450** | **500** | **10** | **IAV=5.732 copies/ 5** µL |
|  |  |  |  |  | **IBV=5.804 copies/5** µL |

For dPCR tests, the fully automated nanoplate-based system, which integrates partitioning, thermocycling, and imaging, the QIAcuity One dPCR System (Qiagen, Hilden, Germany) was used. The QIAcuity OneStep Advanced Probe Kit (Qiagen, #250132) dPCR 8-well nanoplates with a partition capacity of 26,000 partitions were used for dPCR tests.

The reaction mixture was prepared in accordance with the dPCR optimizing protocol of the manufacturer. We used IAV and IBV primers and probes specific to each target as described in CDC FluSc2 multiplex assay (https://archive.cdc.gov/www_cdc_gov/coronavirus/2019-ncov/lab/multiplex.html). For IAV, two forward primers: InfA For1 (5'-CAA GAC CAA TCY TGT CAC CTC TGA C-3'), InfA For2 (5'-CAA GAC CAA TYC TGT CAC CTY TGA C-3'), and two reverse primers: InfA Rev1 (5'-GCA TTY TGG ACA AAV CGT CTA CG-3'), and InfA Rev2 (5'-GCA TTT TGG ATA AAG CGT CTA CG-3') were used. The IBV primers included a forward primer: InfB For (5'-TCC TCA AYT CAC TCT TCG AGC G-3') and a reverse primer InfB Rev (5'-CGG TGC TCT TGA CCA AAT TGG-3'). The IAV probe sequence was 5’-/FAM/TGC AGT CCT /ZEN/ CGC TCA CTG GGC ACG/3IABkFQ/-3’, while the IBV probe sequence was 5’-/YakYel/CCA ATT CGA/ZEN/ GCA GCT GAA ACT GCG GTG/3IABkFQ/-3’.

The total volume of each dPCR reaction was 40 μL for IAV and IBV which included a 10 μL of 4× OneStep Advanced Probe Master Mix, 0.4 μL of 100× OneStep RT Mix, 5 µL of Enhancer GC, primer-probe mix for IAV and IBV with final concentrations of 0.4 μM for the forward and reverse primers and 0.2 μM for the probe. Additionally, 5 μL of positive control template (D4 dilution) and the remaining volume adjusted with RNase-free water. The PCR mix was prepared in an 8-well strip and subsequently transferred to the 8-well nanoplate with a partition capacity of 26,000 partitions. This quantification was performed in duplicates for both non-template control (NTC) and D4 dilution.

Digital PCR was conducted in accordance with the manufacturer’s protocol for reverse transcription at 50°C for 40 minutes followed by PCR enzyme inactivation at 95°C for 2 minutes. The PCR proceeded with 40 cycles of denaturation at 95°C for 5 seconds and combined annealing/extension at 56°C for 30 seconds, with temperature and cycle settings adjusted based on assay requirements.

The DNA copy number for each reaction was obtained directly from the QIAquity software suite version 2.2.0.26 using the Poisson error algorithm and the total error from a random distribution. The well-partition signals and copies/μL results were generated based on the auto-threshold from the software and exported as excel file as the average copies/μL (Tables C and D).

**Table C: dPCR well partition signal results of duplicate reaction of dilution 4 (D4) of IAV and IBV plasmid DNA**

|  |  | IAV target | | | IBV target | | |
| --- | --- | --- | --- | --- | --- | --- | --- |
| Sample | **Replicate (R1/R2)** | **Partitions (valid)** | **Partitions (positive)** | **Partitions (negative)** | **Partitions (valid)** | **Partitions (positive)** | **Partitions (negative)** |
| NTC | R1 | 25294 | 0 | 25294 | 25294 | 0 | 25294 |
| NTC | R2 | 25339 | 0 | 25339 | 25339 | 0 | 25339 |
| D4 | R1 | 25471 | 2581 | 22890 | 25465 | 2627 | 22838 |
| D4 | R2 | 25439 | 2519 | 22920 | 25436 | 2534 | 22902 |

**Table D: dPCR copy number results of duplicate reaction of dilution D4 of IAV and IBV plasmid DNA**

| Sample ID | Replicate (R1/R2) | IAV (copies/µL) | IBV (Copies/µL) | Mean - IAV (Copies/µL) | Mean - IBV (Copies/µL) | IAV (Copies/40µL) | IBV (Copies/40µL) |
| --- | --- | --- | --- | --- | --- | --- | --- |
| NTC | R1 | 0 | 0 | 0 | 0 | 0 | 0 |
| NTC | R2 | 0 | 0 |  |  |  |  |
| D4 | R1 | 144.6 | 147.4 | 143.3 | 145.15 | 5732 | 5806 |
| D4 | R2 | 142 | 142.9 |  |  |  |  |

### **Assay linearity and limit of detection (LOD)**

For the generation of the standard curve, reverse transcription-quantitative polymerase chain reaction (RTqPCR) assay was used for quantifying IAV and IBV DNA. The limit of detection (LOD) of the IAV and IBV RT-qPCR method was evaluated with a dilution series of plasmid DNA using 10-fold serial dilutions from dilution 1(D1) to dilution (D7). The RT-qPCR assays were run in triplicate for D1 to D7 in a total volume of 15 μl.

The reaction mixture was prepared according to the Luna Probe One-Step RT-qPCR optimizing protocol. The same forward and reverse primers and probes specific to IAV and IBV were used as in dPCR, with final primer concentrations at 0.4 μM each and probe concentrations at 0.2 μM each. Each reaction was setup at a volume of 15 μL, containing 3.75 μL of 4× Luna Probe One-Step RT-qPCR Mix with UDG, 0.6 μL each of the forward and reverse primers (For 1, For 2, Rev 1, and Rev 2), 0.3 μL of probes for both IAV and IBV, and 5 μL of template. The reaction mixture was gently mixed by pipetting and briefly centrifuged to collect liquid at the bottom of the tubes. The prepared assay mix was dispensed into qPCR tubes or plates, which were sealed with optically transparent caps or film, ensuring no air bubbles and preventing evaporation.

The qPCR was conducted using the Applied Biosystems - QuantStudio™ 5 Real-Time PCR System. The cycle conditions were 25°C for 30 seconds for carryover prevention, 55°C for 10 minutes for reverse transcription, followed by an initial denaturation at 95°C for 1 minute, and 45 cycles of 95°C for 10 seconds and 56°C for 1 minute for annealing, detection, and extension. The passive reference dye was set to ROX.

Each reaction was performed using the defined protocol for assessing linear relationships and detection limits. The amplitude fluorescence data of the non-template control (NTC) was clean and performed in triplicates. The data generated from RTqPCR exhibited excellent precision (Fig. A). The linearity of the reaction based on linear regression analysis resulted in correlation coefficient R^2^ values of 0.999 for IAV and 0.998 for IBV were observed for dilutions D1 to D6 (Fig. B). However, when analysis was extended to D7, the R² values declined to 0.924 for IAV and 0.916 for IBV, suggesting a less reliable linear relationship. Therefore, to maintain optimal linearity, we defined the LOQ at the D6 dilution level, with copy counts of 11.46 copies/μL for IAV and 11.61 copies/μL for IBV, where the coefficient of variation (CV) remained consistently below 15%, meeting the criteria for accurate quantification.

To determine the limit of detection (LOD), additional analyses were conducted using two-fold dilution from D6, each dilution with IAV and IBV plasmid positive controls were tested in 20 replicates in RT-qPCR. The D7 dilution achieved a 95% detection rate among replicates, establishing a 95% confidence interval (CI), whereas D8 did not meet the 95% CI threshold. Thus, the LOD for this assay was determined to be the D7 dilution, where reliable detection within the desired confidence interval was achieved. The LOD at D7 was 5.73 copies/µL for IAV and 5.8 copies/µL for IBV and D8 2.87 copies/μL for IAV and 2.90 copies/µL for IBV.

With the standard curve generated from the RT-qPCR assay, wastewater isolated RNA samples tested in duplicates, CT values obtained were interpolated and quantified for both IAV and IBV.


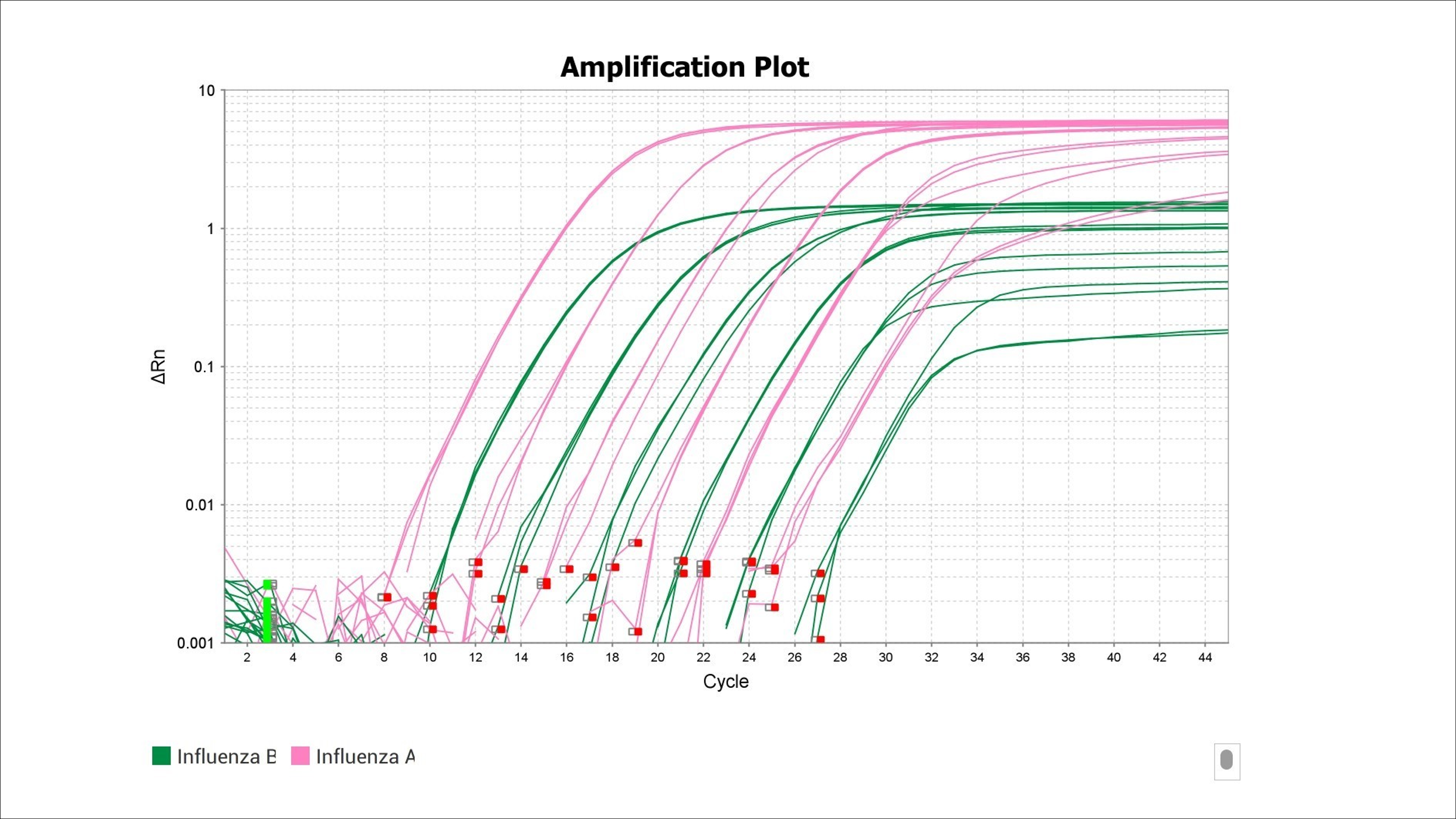


Fig. A Amplification plots for IAV plasmid DNA using 10-fold serial dilutions from dilution 1(D1) to dilution (D6) in triplicates.


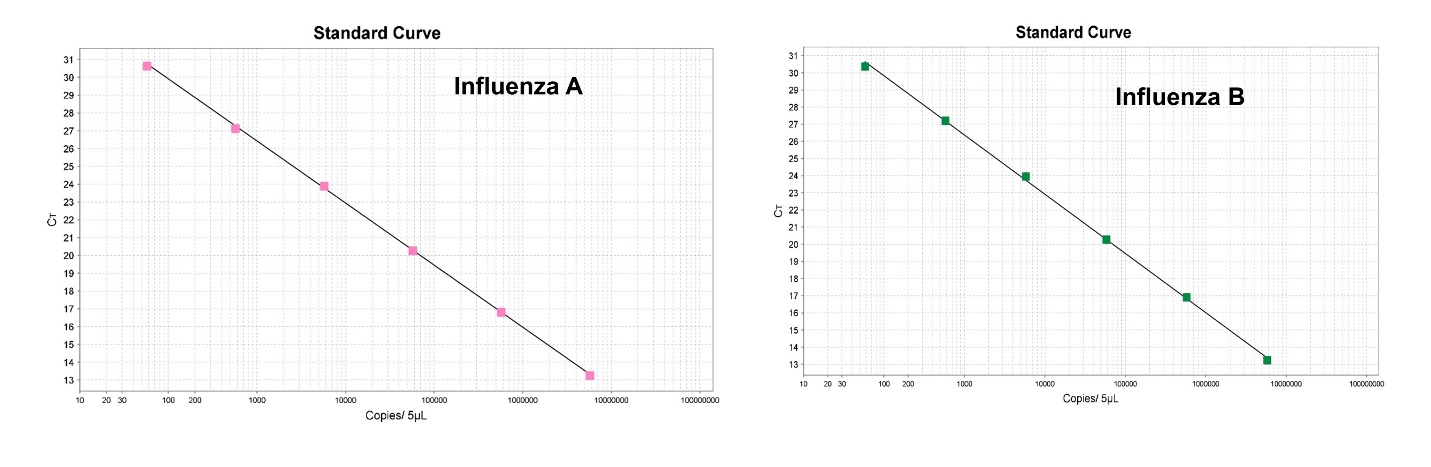


Fig. B. Standard curve for IAV and IBV plasmid DNA using 10-fold serial dilutions from dilution 1(D1) to dilution (D6) in triplicates.

**Formula used for the quantification of IAV and IBV number of copies/µL in samples tested obtained from the linear standard curve generated**

| **Number of copies/ µL of IAV in samples tested                              =** | **10^ [(Ct value of samples tested – Y-intercept)/(Slope)]/Volume of sample used in RT-qPCR in µL** |
| --- | --- |

| **Number of copies/ µL of IAV in samples tested                              =** | **10^ [(Ct value of sample tested – 36.888)/ (-3.487)]/5** |
| --- | --- |

| **Number of copies/ µL of IBV in samples tested                              =** | **10^ [(Ct value of samples tested – Y-intercept)/(Slope)]/ Volume of sample used in RT-qPCR in µL** |
| --- | --- |

| **Number of copies/ µL of IBV in samples tested                              =** | **10^ [(Ct value of sample tested – 36.729)/ (-3.454)]/5** |
| --- | --- |

**IAV and IBV viral load quantification**

**Formula used for quantification of IAV and IBV number of copies/mL of wastewater in samples tested**

| Number of copies of target / mL of wastewater in samples tested = | [(Number of copies of target/ µL of samples tested x RNA Elution volume in µL x Total lysate volume used for pellet resuspension from 40mL of wastewater processed)/ Lysate volume used for RNA extraction]/ Volume of wastewater samples processed in mL |
| --- | --- |

| **Number of copies of IAV / mL of wastewater in samples tested =** | **[(Number of copies of IAV / µL of samples tested x 50 µL x 600 µL)/140 µL]/ 40 mL** |
| --- | --- |

| **Number of copies of IBV / mL of wastewater in samples tested =** | **[(Number of copies of IBV/ µL of samples tested x 50 µL x 600 µL)/140 µL]/ 40 mL** |
| --- | --- |
