# Supplementary figures and images for: Respiratory virus infection dynamics and genomic surveillance to detect seasonal influenza subtypes in wastewater: A longitudinal study in Bengaluru, India
Source: PLOS Glob Public Health. 2025 Sep 12;5(9):e0004640. doi: 10.1371/journal.pgph.0004640 (PMC12431432; doi:10.1371/journal.pgph.0004640)

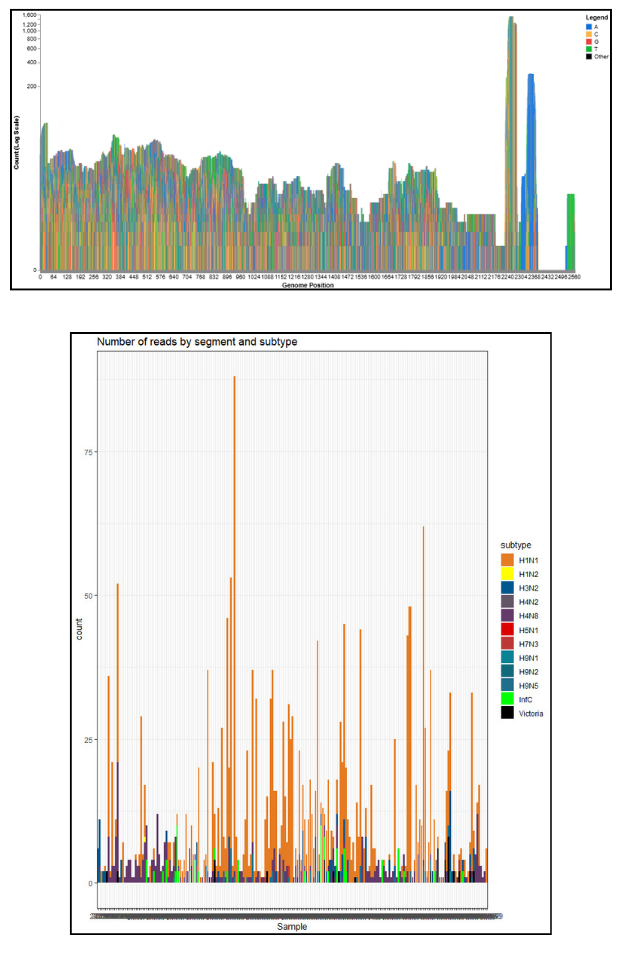

Supplement: S1 Fig — Panels show dereplicated reads from virome sequencing aligned to influenza A genomes in the NCBI database and the distribution of influenza subtype reads across samples. (PNG) [file pgph.0004640.s008.png]
